# Transverse pinning of concomitant first and second metatarsal fractures using 1.5mm K-wires; case report and technical note

**DOI:** 10.1016/j.amsu.2022.103906

**Published:** 2022-06-08

**Authors:** Alireza Moharrami, Seyed Peyman Mirghaderi, Nima Hoseini Zare, Seyed Pouya Tabatabaei Irani, Mir Mansour Moazen-Jamshidi, Seyed Hadi Kalantar

**Affiliations:** a Joint Reconstruction Research Center, Tehran University of Medical Sciences, Tehran, Iran; b Students' Scientific Research Center (SSRC), Tehran University of Medical Sciences, Tehran, Iran

**Keywords:** Bone wires, Case report, Metatarsal bones, Transfixation, Transverse pinning

## Abstract

**Introduction and importance:**

Here we represented a new technique of closed reduction and transverse pinning to address first metatarsal comminuted fractures in patients with a concomitant second metatarsal shaft fracture.

**Case presentation:**

The first metatarsal comminuted fracture coincides with the second metatarsal simple fracture in this forefoot injury case. In a new technique, we used close reduction and percutaneous pinning **(**CRPP) in a transverse direction of pins to achieve a satisfactory outcome.

After performing traditional CRPP to fix the second metatarsal fracture, it served as physical support for the first metatarsal fixation. We drilled two 1.5mm pins through the first metatarsal bone at each proximal and distal side of the fracture site, transversely passed to the second metatarsal bone. Transverse pins came along from the first metatarsal medial side to the lateral. After six-week and 12-month follow-up, the patients had minimal pain with complete radiological and clinical fracture healing and no complication.

**Clinical discussion:**

Here, internal fixation was unsuitable due to extensive soft-tissue injury and inadequate bone support. Despite the many advantages of external fixators, they have drawbacks that persuade us to perform our new technique: using K-wires for transverse pinning fixation of the first metatarsal fracture using an adjacent metatarsal as support. This minimally invasive approach is profitable because of its minimal soft tissue damage, affordable price, and convenient access.

**Conclusion:**

The transfixation technique with K-wires is rarely used to treat metatarsal fractures. It may be helpful in similar cases of comminuted first metatarsal fracture with satisfactory outcomes.

## Highlights

• We introduced an applicable method for fixing comminuted first metatarsal fracture.
• Transverse pinning has appropriate clinical and radiological healing.
• Transfixation technique with K-wires is rarely used to treat metatarsal fractures.

## 1 Introduction

Metatarsal fractures are among the most common injuries to the foot, accounting for 35% of all foot fractures and 5–6% of fractures visited in primary care. As the first metatarsal bone bears weight two times more than other metatarsals, it has a crucial role in the foot stability and gait. First metatarsal fractures occur after direct trauma (more common in industry injuries) or indirect forces (twisting injury in sport) [1]. Although First metatarsal fractures are infrequent- 1.5% of all metatarsal fractures-they must be renovated skilfully because of the severe morbidity [2,3].

Intact anatomy and acceptable length of the foot's medial ray play a crucial role in a normal gait; Because it bears about 40% of body weight in the stance phase. The first ray also acts as a lever arm for the Achilles tendon to transfer force to metatarsals during the gait's propulsive phase [4]. As a debilitating problem after metatarsal fracture, residual mal-alignment and malunion of the first metatarsal fracture lead to metatarsalgia and anatomical deformity in the first ray [5,6]. Shortening the medial ray is one of the typical deformities that may cause major complications. Cavus foot is a complication of a shortened medial ray [7]. Also, secondary to unequal weight-bearing of the forefoot, transfer lesion of lesser metatarsal followed by MTP synovitis and rupture of collateral ligaments can result in transfer metatarsalgia [1,4]. Another common deformity is dorsal angulation of the distal part. Malunion is the consequence of inappropriate fixation intraoperatively or improper execution of post-op precautions. Thus, foot injury treatment must consider the appropriate length of medial ray and precise aligning, which is an arduous job in comminuted fractures [4].

The pattern of fracture, stability and displacement level determine optional treatment. Instead, displacement and instability of fracture need aggressive reduction and immobilization, which indicates surgical treatment. Surgeons use a range of fixation techniques depending on fracture configuration [7]. In unstable shaft fracture of the first metatarsal with significant displacement, open reduction and internal fixation (ORIF) is the choice of care (buttress plating with screw fixation) [7,8]. Simple diaphyseal fractures are fixed with a lag screw, whether oblique or spiral. A one-third tubular bridging plate or a 2.7-mm plate is applied when more stability is required to fix a more comminuted fracture [1,7]. If a severely comminuted fracture occurs, the internal fixation technique is no longer an option due to the sufficient bone support. Therefore, external fixation is the choice. External fixation is also a proper procedure to conserve soft-tissue integrity and repair as long as fixing the fracture [1,7]. In another case report study, Ilizarov mini external fixator was used to fix a first metatarsal comminuted fracture [9].

Despite advantages, the external fixator has its blind spots. Patients are uncomfortable with the external fixator because of its bulky and cumbersome frame. Furthermore, fracture at the hole sites is probable once the rods are removed [10]. Setting up an external fixator during operation is challenging and needs more monitoring after installation [11]. The high cost of the device and less accessibility in developing countries than pinning techniques are other drawbacks. The external fixator was unavailable for us in our academic center. Therefore, we used the percutaneous pinning technique, unlike the literature prefer. In a new technique for stabilizing the comminuted fracture of the first metatarsal shaft, accompanied by a simple shaft fracture of the second metatarsal, we employed close reduction and percutaneous pinning **(**CRPP) in transverse direction for first metatarsal fracture, fixed to the second metatarsal as the support. The transfixation technique with K-wires is rarely used to treat metatarsal fractures. In this technique, we observed proper healing and union of fracture with no limiting complications, thus it may be helpful in similar cases of comminuted first metatarsal fracture. The Surgical CAse REport (SCARE) Guidelines were followed in reporting this study [12].

## 2 Presentation of case

A 40-year-old white man without past medical history presented to the emergency service of our center (Imam Khomeini Hospital, Tehran, Iran) with a closed forefoot fracture after sustaining a direct trauma with a heavy object. The patient has ecchymosis and soft tissue swelling on the dorsal of his foot, tenderness, and deformity in the medial column, and was unable to weight-bearing. No family history of illness or use of medications has been reported.

**Fig. 1:**
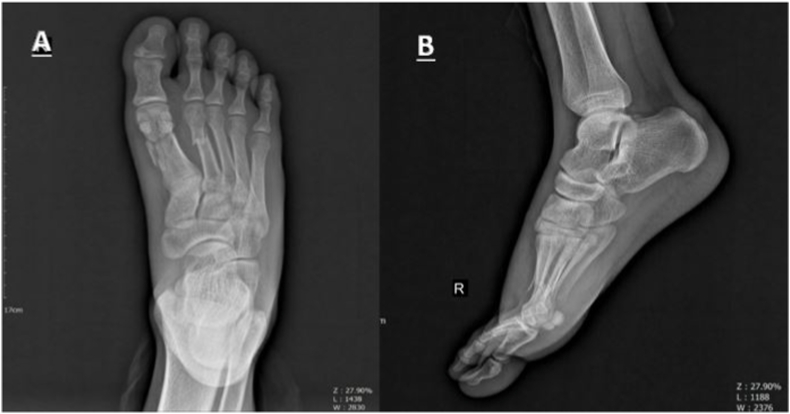
**A**, Anteroposterior view of initial injury presenting a Comminuted fracture of the shaft of the first metatarsal and a simple fracture of the second metatarsal shaft with displacement **B**, Lateral view.

**Fig. 2:**
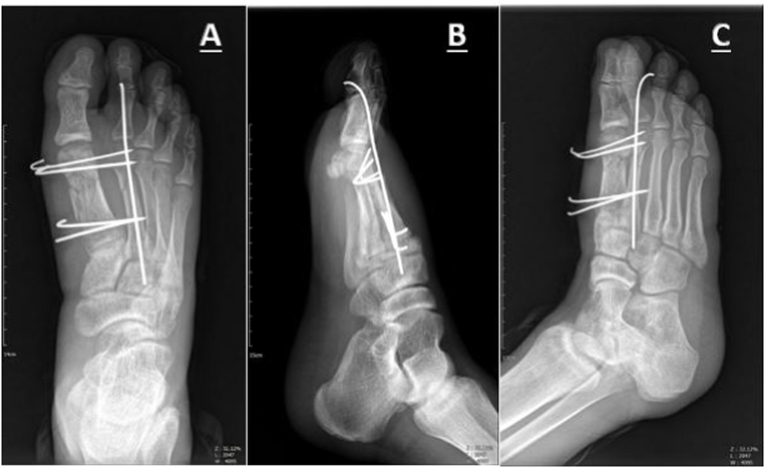
Proper alignment and stability of first and second metatarsal after close reduction and internal fixation with 1.5mm pin **A**, Anteroposterior view **B**, Lateral view **C**, oblique view.

Radiography of the foot revealed a close concomitant Comminuted fracture of the shaft of the first metatarsal (OTA.87.1.2C) and a close transverse simple fracture of the second metatarsal (OTA.87.2.2A) of the right lower limb in our patient (Fig. 1). He underwent surgery with a new technique (detailed below). The fracture was fixed with one longitudinal 1.5 mm K-wire for the second metatarsal, and the first metatarsal was transfixed with four 1.5 mm K-wires (Fig. 2).

**Fig. 3:**
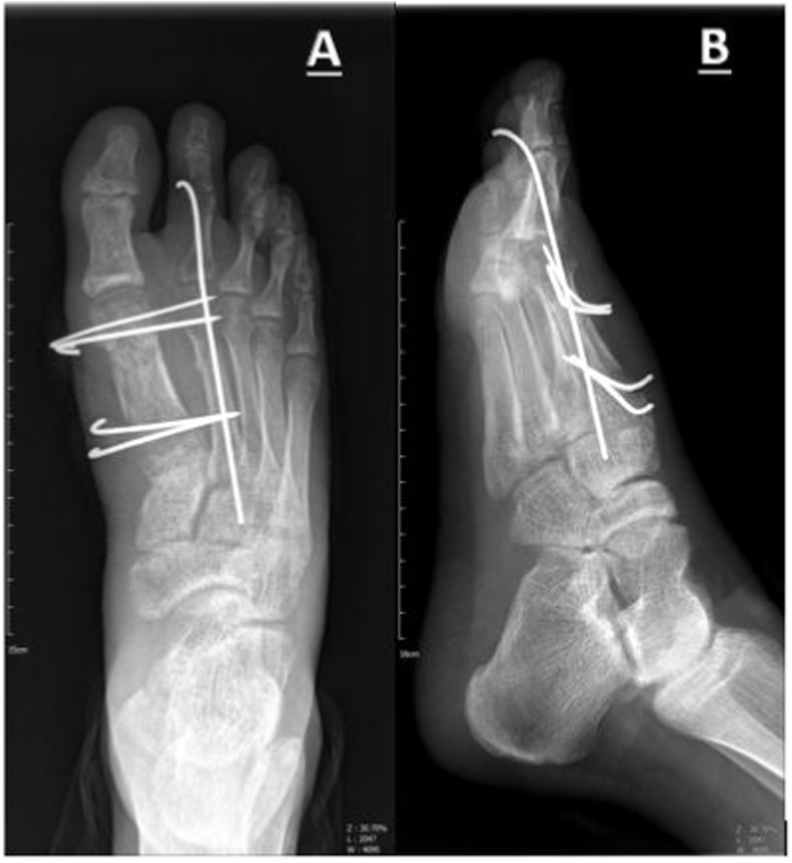
Proper union and callus formation at 6-week follow-up visit **A**, Anteroposterior view **B**, Lateral view.

**Fig. 4:**
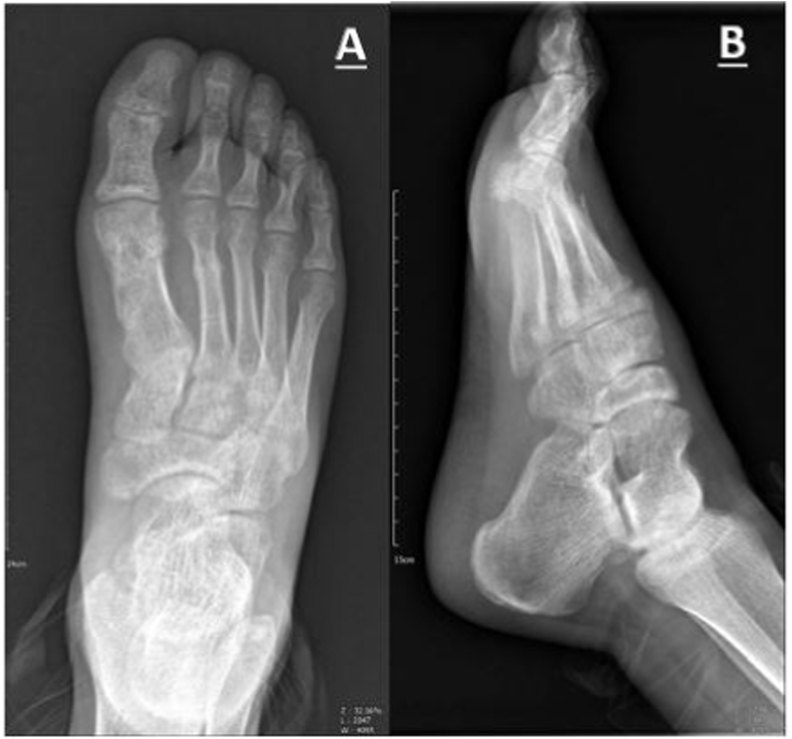
Complete healing of fracture sites at 1-year follow-up **A**, Anteroposterior view **B**, Lateral view.

We kept the K-wires for six weeks with short leg casting, and we opened the cast and pulled out the pins when radiographic evaluation confirmed the union of fracture sites (Fig. 3). At the 12-month follow-up, the patient had no complaints of pain in his foot, and he achieved full function, weight-bearing, and range of motion (ROM) in his last follow-up (Fig. 4). He returned to his work as a salesperson after two months. The AO foot and ankle score (AOFAS) was measured for the patient that showed an excellent score (97/100).

## 3 Technique

The surgery was performed by the senior resident (A.M, PGY-3) under the observation of the senior author, who is an assistant professor of orthopedic and trauma surgery (SH·K). We performed the surgery with the patients under spinal anesthesia and on a radiolucent table in a supine position. We put bolster support under the ipsilateral hip and the injured foot in a neutral position. The intramedullary fixation was carried out using Kirschner wire of 1.5 mm diameter and single-ended.

**Fig. 5:**
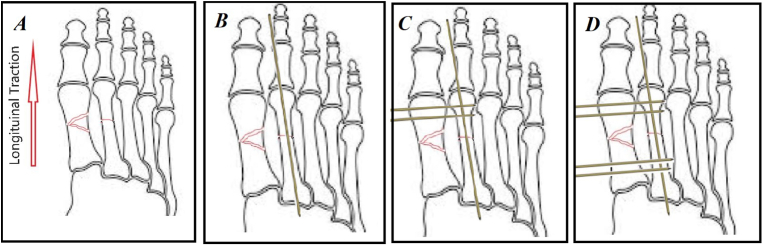
In our technique, we firstly applied longitudinal traction to the first and second metatarsal (A), then we performed a 1.5 mm intramedullary K-wire in the second metatarsal (B), and then we applied 2 1.5 mm transverse K-wire distal to the fracture from first to the second metatarsal (C) and finally 2 1.5 mm K-wire applied in the proximal of the fracture transversally (D).

For fixation of the second metatarsal, we performed closed reduction using longitudinal traction (Fig. 5A) and applying a 1.5mm intramedullary pin (Fig. 5B). The K-wire was inserted antegrade, proximal to the fracture, into the distal medullary canal. In the following procedure step, the K-wire was drilled through the metatarsal head while its sharp edge protruded from the foot's plantar skin. On the opposite end of the K-wire, a sharp cut was made; the drill was then disconnected from its proximal part and connected to its distal part in the plantar surface of the foot. Drilling the wire into the fracture site was then performed. Following proper fracture reduction with the aim of the inserted K-Wire, longitudinal toe traction, and manipulation, the K-Wire was retrogradely introduced to the proximal segment of bone. An intraoperative C-arm fluoroscopic image was used to verify reduction and K-Wire positioning. Further drilling of the KW was conducted until it reached the metatarsal base of the medullary canal. An end of a wire protruding from the plantar surface was trimmed and bent.

After the second metatarsal fixation, it was exploited as physical support for the first metatarsal. Then, we drilled two 1.5mm pins at the head (Fig. 5C) and two 1.5mm pins at the base of the first metatarsal transversely passed to the second metatarsal bone (Fig. 5D). Pins were placed approximately 10 mm far from the fracture site to avoid the articular surfaces. Transverse pins were advanced from the medial side of the first metatarsal to the lateral. We observed stability of reduction by C-arm fluoroscopic imaging to check the outcome. Proper alignment accompanied by stability maintenance was achieved after this procedure.

## 4 Postoperative care and follow up

After discharge, the patient was mobilized with no weight-bearing and ankle pumping after a day to prevent deep vein thrombosis and ankle stiffness. At first, 3, and 6 weeks, follow-up visits showed no signs of ecchymosis or pin site infection but only mild pain. In the first week's visit, we applied short leg casting for the patients after confirming no sign of swelling and compartment syndrome risk. Proper union and callus formation was established in the radiograph in the sixth week (Fig. 3). Hence, pins were pulled out, and weight-bearing was started on the foot as tolerated. At a mean of 12-months follow-up, we observed complete radiological and clinical union without any tenderness, residual pain, or complications in all the patients. The radiograph illustrated complete healing of the fracture site with correct alignment and length of the first metatarsal (Fig. 4). The patient had a full range of motion and normal strength in his foot. The American Orthopedic Foot and Ankle Society (AOFAS) score was measured for the patients that showed the mean of the high score (98/100). The AOFAS scoring system consists of three subjective and objective parameters, including pain (40 points), function (50 points), and alignment (10 points). There were no reports of residual pain. The patient declared he completely adhered to medical advice and rehabilitation exercises instructed by the doctor.

## 5 Patient perspective

He stated that he was satisfied with the surgery and treatment in the latest follow-up. Upon examination, the patient declared he had no discomfort and no functional impairments.

## 6 Discussion

The first metatarsal traumatic malfunction and disintegration can cause noticeable morbidity and disturb physiologic gait [2,3,7]. A medial ray's substantial role in gait and weight-bearing causes patients to face a dilemma (shortening, malreduction, etc.). A medial ray mal-alignment and post-traumatic deformity can result in chronic foot pain and metatarsalgia, Cavus foot deformity, transfer injury of the lesser metatarsal, hallux valgus, and degenerative arthritis. In the end, treatment aims to restore the normal anatomy to prevent such complications.

Treatment options are determined by the fracture pattern and degree of displacement. In this case, a severe Comminuted fracture of the first metatarsal shaft occurs in conjunction with a transverse simple shaft fracture of the second metatarsal. We decided not to use ORIF due to extensive soft-tissue injury and inadequate bone support. Thus, an external fixator is a preferred treatment plan to fix these fractures while ensuring good soft tissue repair [1,7]. Despite their many advantages, external fixators have a few drawbacks. External fixators require precise monitoring postoperatively, have a higher risk of damaging the neurovascular system than pin placement, a possibility of infection, and cause daily activity disturbance due to the bulky frame [11]. There have been discussions that external fixator devices have a high cost and are difficult to access in developing countries.

This is the first article to report the use of K-wires for transverse pinning fixation of the first metatarsal fracture using an adjacent metatarsal as support. Its minimally invasive approach turns a profit due to minimal soft tissue damage, affordable price, and convenient access. However, pin-tract infection threat always exists [8]. As far as advantages and complications are concerned, we used the percutaneous pinning technique for comminuted fractures and displacement of the first metatarsal.

Transverse pinning is a well-established technique in the fixation of metacarpal fractures with significant angulation/displacement [[13], [14], [15], [16]]. The procedure is straightforward and rapid, provides reliable stability, and can be used to treat a single metacarpal shaft, neck, and base fracture [17,18]. Compared to ORIF, percutaneous pinning has a lower complication rate, including infection, fracture non-union, and soft tissue damage since no soft tissue is dissected. Removing hardware can also be done without anesthesia, thus lowering treatment costs [19]. It is also possible to re-insert the pin if the placement is unsatisfactory [18].

In a case of a displaced lateral metatarsal neck fracture, Donahue MP et al. used the CRPP technique with pins transversely fixed to nearby intact metatarsal bones. They suggest this technique can be beneficial, especially in multiple metatarsal neck fractures regarding lower soft-tissue insult [20]. This technique may be logical in conditions such as low soft-tissue support, no access to external fixators, and multiple metatarsal fractures. Concerns still exist regarding the level of experience that surgeons should have. Future research can shed light on the effectiveness of this method.

In conclusion, Closed reduction followed by transverse pinning for the treatment of first metatarsal fractures has appropriate clinical and radiological healing. It can be a promising fixation method in patients with comminuted first metatarsal fractures with low soft tissue support. Further studies on a larger population of patients with more long-lasting follow-ups are necessary to distinguish the effectiveness and safety of this method.

## Ethical approval

N/A.

## Sources of funding

There is no source of funding for the research.

## Author contribution

Author 1: case report concept and design, data collection. Author 2: writing the paper and literature review. Author 3, 4, and 5: surgical intervention, patient consent, and data collection. Author 6: design, editing, and reviewer.

## Consent

Written informed consent was obtained from the patient to publish this case report and accompanying images. A copy of the written consent is available for review by the Editor-in-Chief of this journal on request.

## Research registration

N/A.

## Provenance and peer review

Not commissioned, externally peer reviewed.

## Declaration of competing interest

The authors report no declarations of interest.
